# Progress in Medicine

**Published:** 1895-03-23

**Authors:** 


### PROGRESS IN MEDICINE.

### DISEASES OF THE CIRCULATORY SYSTEM.

**Treatment.**—The waters of the Llangammarch springs,[1] in Brecon, have been brought into prominence as a heart tonic. They contain 6½ grains to the gallon of barium chloride. The therapeutic dose is about two tumblerfuls containing one grain of the chloride, and the action appears to be much that of digitalis, producing increased and slower action of the heart, with contraction of the vessels. Useful results have been found in mitral disease, acute dilatation, and functional derangements, as well as in glandular swellings. Bezly Thorne[2] regards their effect as similar to that of the Nauheim waters, and has found them of great value in the treatment of dilatation of the heart and anæmia, as well as for glandular swellings. F. George points out that the barium chloride is not alone, but has with it full doses of calcium chloride, and believes that the chief value of the waters will be found to be in strumous diseases. [We may refer to the remarkable effects of calcium chloride in chronic phthisis, gastric disorders, and enlarged glands, noticed by Sawyer, Germain See, and Begbie.] He thinks the ordinary dose should be one tumbler three or four times a day. The Llangammarch springs[3] fortunately contain little or no carbonic acid gas, which has an injurious effect when mingled with barium salts, rendering soluble the poisonous barium carbonate.

Discussion has been continued on the Schott treatment by saline baths at a temperature of 87 deg. to

93 deg., with regulated exercise movements. Babcock[4] considers the effect of the baths is to stimulate, slow, and strengthen the contractions of the heart, while they dilate on the whole the vessels, and thus reduce rather than increase the peripheral resistance. The gentle movements of the limbs, with counter-resistance applied by an attendant, also increase the force and volume of the pulse. He regards them as counter-indicated in advanced arterio sclerosis, aneurism, acute softening, or great fatty degeneration. However, Schott appears to find that cases of moderate fatty degeneration, together with those of dilatation, whether valvular lesions are present or no, receive much benefit from the treatment. Wethered thinks[5] the method valuable in angina pectoris, which is, however, denied by Huchard. Emyly[6] calls attention to the increased diuresis, the diminished cardiac dulness, and increased force and slowness of the heart, with relief of dyspnœa and œdema, and concludes that these results are due to (1) increased arterial circulation with diminished peripheral resistance, (2) diminished venous congestion, and (3) diminished work for the heart. The regulated movements produce a great part of the result, and these can be undertaken at home.

Paganstecher[7] gives an analysis of these movements which are derived from the Swedish system of gymnastics. An important point is that the exercise should be stopped the moment the breathing is quickened, and no *séance* should exceed thirty minutes. H. Campbell[8] points out that in treating mitral disease the first thing is to promote the pulmonary circulation and to relieve the right heart by encouraging the respiratory movements in every possible way. The second accessory force of the circulation consists in muscular contractions, which drives on the blood in the veins, and the lymph in the lymphatics. Excessive action, however, leads to fixation of the thorax and actual hindrance to the pulmonary and venous circulation. Hence the great value of regulated movements. Symons Eccles[9] lays down a system of treatment for irregular dilated hearts. It commences with complete rest in bed for two or four weeks with gently increased massage, followed by passive and then by active resisted movements. These must be attempted very gradually and in a methodical manner, and finally walking exercise is taken by degrees and most carefully limited.

**Cardiac Tonics.**—Cerna has investigated the physiological action of sparteine,[10] and finds it causes a stimulation of the irritability of the peripheral nervous system, which is followed by a distinct depression, but there is no proof that it acts as a narcotic. A noticeable feature is the great increase in size of the individual pulse waves probably produced by its action on the heart muscle itself. In excessive doses it kills by respiratory failure.

Pavinsky[11] recommends a combination of caffeine with benzoate or salicylate of soda both as a diuretic and in a failing heart where digitalis and strophanthus have lost their power. The drug appears to act rather on the vaso-motor centres than on the cardiac nerves, and in cases of shock or collapse it has a special value. Apocynum cannabinum[12] has been a good deal used for cardiac dropsy, and one preparation (the decoction) has emetic and cathartic effects. The tincture contains an amorphous principle (apocynin), and causes great diuresis without irritating the kidneys or stomach. The arterial tension is not constantly increased, and the drug may be used for a long while. Glinski[13] claims that it diminishes the area of dulness in dilatation and stops the palpitation, while it has no cumulative action and has great diuretic powers. Kemmerich writes on the effect of meat peptone. Under its administration the rate of the pulse is slowed and the contractions become more forcible. This can be observed about half an hour after the peptone has been taken, and a mild diuresis is also produced. The "Fleischpepton,"[14] with which he experimented, contains albumoses 30 per cent., peptone 18, other albuminous bodies 9 or 12 per cent., besides the extractives and phosphates. The discussion on the alkaloids of digitalis has been continued by Masius.[15] He claims that German digitoxine has the same therapeutic powers as digitalis, and prefers it to the French crystalline digitaline and digitaline verum. He gives one sixty-fourth of a grain divided into three doses daily. The gastric disturbances are few and unimportant. Its powerful effect on the pulse and diuresis may last eight or ten days, and in typhoid and pneumonia there is a marked effect on the temperature.

Van Aubel[16] proposes the intravenous injection of digitoxine and strophanthin in doses of half a milligramme of digitoxine and one-tenth milligramme of strophanthin, which he believes will be of service in emergencies. A denial of the cumulative action of digitalis by Beaumont Small is warmly opposed in the *Therapeutic Gazette.*[17] The editor asserts that it may be given without effect for two days, and then exert its influence suddenly, and also that its influence may continue for a week after administration. Again, if given in ascites or fever, it produces an excessive effect when these conditions have passed away. An overdose may cause forcible beating of the heart, with no radial pulse, or remarkable slowness alternating with rapid action, or even a condition in which the first sound of the heart is inaudible. This fear of the effect of the drug seems to have more supporters in America than in this country, and to be widely accepted there.

Cactus Grandiflorus has been tried by Gordon Sharp,[18] who failed to find in it either alkaloid or glucoside. The resins contained in it have, however, a residue which reduces Fehling's solution. No action like that of digitalis on the frog's heart could be discovered, nor in healthy persons did very large doses produce any result. In the cases of ascites on which he tried it, digitalis seems to have had, however, no better action. He was unable to demonstrate the power claimed for it of shortening the cardiac diastole, and thinks that all that can be said for it is that it has a slight diuretic action. Mikhailoff,[19] on the other hand, finds it produces a definite but transient rise of tension, while palpitation and dyspnœa were quickly stopped by it. He considers that it must be given for a long time in increasing doses to effect much permanent improvement. Huchard, in commencing œdema from cardiac asystole, keeps his patients carefully in bed on a milk diet for some days, and then, after a strong purgative, he administers a single massive dose of 40 or 50 drops of 1 in 1,000 solution of crystallised digitaline, which causes copious diuresis.[20] Potain agrees in the practice of giving a single large dose. In some cases Huchard gives a dose of digitaline once a fortnight, and theobromine on three days in addition.[21] Panowski reports on diuretin that it reduces the area of cardiac dulness by acting on the heart muscle,[22] and causes diuresis only by stimulation of the nerve centres and the consequent rise of pressure. He uses it instead of digitalis where the pulse is very slow, as in uræmic states, and when digitalis has failed. H. A. Hare points out that where hypertrophy[23] had definitely occurred during an active life, and palpitation and irregularity followed after a more sedentary one had been taken up, it is a mistake to use such drugs as digitalis. In place of it he employs a sedative, such as aconite, gelseminum, or veratrium viride. The nitrites and atropine are really sedatives of the same kind. Sedatives are indicated where the heart beats with at least sufficient force, but spasmodically, irregularly, and ineffectually. In a careful paper on the treatment of organic heart disease, Beverley Robinson[24] mentions the value of small and long-continued doses of digitalis, with or without iron and strychnia, for dilatation in the course of mitral disease. In late stages, when dropsy has set in, he would combine with it nitro-glycerine, and, if the œdema is extreme, he adds cathartics and strictly limits the amount of fluid taken. In tricuspid failure we find cases where venesection affords great and rapid relief, even if old emphysema is present. Caffeine is of great value, especially when much dyspnœa occurs in mitral stenosis, and also in aortic regurgitation and when digitalis has failed. Convallaria, too, occasionally is useful in mitral stenosis and other cases. In stenosis the nitrites and nux vomica must not be neglected. He prefers the bromides to aconite for the class of cases in which Hare recommends the latter, and combats his exaggerated view of the danger of digitalis. In aortic lesions we do not often enough use belladonna and the iodides, which are capable of giving great relief, especially where the pain is due to a lack of synchronism between the two sides of the heart with deficient nervous control. In addition to these remedies, he lays stress on the importance of general treatment in combatting the gradual failure of the heart muscle.

[1] Lanc., Nov. 24. [2] Lanc., Dec. 1. [3] Lanc., Dec. 15. [4] Med. Rec., Dec. 8. [5] B.M.J., Nov. 10. [6] Dublin J. Med. Sc., Sept. 1. [7] Am. J. Med. Sc., Nov. [8] B.M.J., Nov. 17. [9] Pract., Aug. [10] Am. J. Med. Sc., Sept. [11] Practit., Aug. [12] B.M.J. Sept. 27. [13] Ther. Gaz., Oct. 15. [14] Am. J. Med. Sc., July. [15] Am. J. Med. Sc., Dec. [16] Med. Week, Nov. [17] Ther. Gaz. Sept. 15. [18] Pract., Sept. [19] B.M.J., Sept. [20] Lanc., Sept. 22. [21] Ther. Gaz., Nov. [22] B.M.J., Nov. 3. [23] Practit., Jany., '95. [24] Am. J. Med. Sc., Dec.

